# Diurnal Preference Predicts Phase Differences in Expression of Human Peripheral Circadian Clock Genes

**DOI:** 10.5334/jcr.ae

**Published:** 2015-06-05

**Authors:** Andrew Ferrante, David Gellerman, Ahmet Ay, Kerri Pruitt Woods, Allan Michael Filipowicz, Kriti Jain, Neil Bearden, Krista Kenyon Ingram

**Affiliations:** Department of Biology, Colgate University, NY USA; Management and Organizations, Johnson School of Business, Cornell University, NY USA; Organizational Behavior and Human Resources, IE Business School, Madrid, Spain; Decision Sciences INSEAD, Singapore

**Keywords:** chronobiology, chronotype, gene expression, circadian clock

## Abstract

**Background:** Circadian rhythms play an integral role in human behavior, physiology and health. Individual differences in daily rhythms (chronotypes) can affect individual sleep-wake cycles, activity patterns and behavioral choices. Diurnal preference, the tendency towards morningness or eveningness among individuals, has been associated with interpersonal variation in circadian clock-related output measures, including body temperature, melatonin levels and clock gene mRNA in blood, oral mucosa, and dermal fibroblast cell cultures.

**Methods:** Here we report gene expression data from two principal clock genes sampled from hair follicle cells, a peripheral circadian clock. Hair follicle cells from fourteen individuals of extreme morning or evening chronotype were sampled at three time points. RNA was extracted and quantitative PCR assays were used to measure mRNA expression patterns of two clock genes, *Per3* and *Nr1d2*.

**Results:** We found significant differences in clock gene expression over time between chronotype groups, independent of gender or age of participants. Extreme evening chronotypes have a delay in phase of circadian clock gene oscillation relative to extreme morning types. Variation in the molecular clockwork of chronotype groups represents nearly three-hour phase differences (*Per3*: 2.61 hours; *Nr1d2*: 3.08 hours, both: 2.86) in circadian oscillations of these clock genes.

**Conclusions:** The measurement of gene expression from hair follicles at three time points allows for a direct, efficient method of estimating phase shifts of a peripheral circadian clock in real-life conditions. The robust phase differences in temporal expression of clock genes associated with diurnal preferences provide the framework for further studies of the molecular mechanisms and gene-by-environment interactions underlying chronotype-specific behavioral phenomena, including social jetlag.

## Introduction

Circadian rhythms, the daily patterns of sleep-wake and activity cycles, are one of the major drivers of human behavior [1]. These rhythms are regulated by an internal molecular clock and driven by negative feedback loops of clock genes that cycle across an approximate 24-hour period [2, 3, 4]. Variation in the timing of individual circadian rhythms is a result of endogenous factors (gene mutations and/or variation in gene regulation) [5], external environmental stimuli [6, 7], or both. Thus, the circadian system is an excellent model for understanding the complex effects of gene-by-environment interactions on human behavior.

Traditionally, studies of the wide variation in individual circadian rhythms have focused on malfunctions in the clockwork that lead to diverse disorders affecting digestive systems, renal function and cardiac function. Short-term circadian-related disruptions are common and include jet lag and negative effects of shift work, but long-term perturbations of circadian rhythms have been linked to serious health issues including sleeping disorders, metabolic and psychiatric syndromes, and cancer [8, 9, 10 11].

Recently, a growing number of psychological studies have focused on how interpersonal variation in circadian rhythms, or chronotypes, influence human behavior [12, 13, 14]. A chronotype measure using the Horne-Ostberg scale [15] represents an individual’s preference for morningness or eveningness, and surveys general lifestyle patterns, such as sleep/wake cycles and peak alertness [16]. Morning chronotypes (larks) rise early in the day and show peak alertness in the mid-morning hours. Evening chronotypes (night owls) tend to be late-risers and exhibit peak alertness later in the day, often late into the evening. Extreme chronotypes (approx. 10% of population) are more likely to present adverse symptoms related to circadian rhythms; for example, increased stress levels in evening chronotypes are related to sleep apnea in obese individuals [17].

Morningness-eveningness chronotypes also show marked differences in a number of diverse behaviors, including task performance and decision-making abilities [18, 19]. Even small disruptions in circadian timing (jetlag or daylight savings time changes) can lead to declines in performance (e.g. traffic accidents [20] or lower stock market returns [21]). The common, modern-day phenomenon of social jetlag (when an individual’s internal and external cycles are not synchronized) underscores the need for a clear understanding of how individual-specific chronotypes behave at particular times of day. Often there is evidence of chronotype-by-situation interactions; morning types are more alert in the morning and evening types are more alert in the evening, and this can affect time-specific performance [22]. For example, morning types have more difficulty with night shift work [6]. Similarly, explicit memory tasks, requiring a high level of controlled processing, are better performed at peak times, e.g. morning for morning types [23]. Ethical decision-making may also be affected by diurnal preference and time-of-day interactions [24]. Dissecting the molecular basis of these chronotype-by-situation interactions requires a clear understanding of the genetic mechanisms driving chronotype-specific differences in human behavior.

A number of studies have demonstrated that morningness-eveningness is associated with physiological markers of circadian rhythms [25]. Oscillations in melatonin levels and body temperatures show phase differences in morning versus evening subjects [26, 27, 28]. However, physiological markers, like melatonin, are affected by light exposure and other environmental factors, and endogenous levels can often vary widely between individuals. Phase differences in clock gene expression can also be detected in blood [29], oral mucosa [30], and dermal fibroblast cell cultures [1] but these require in-laboratory testing procedures (cell cultures) or coping with degraded RNA signals (oral extractions). Due to the difficulty of periodic, direct sampling of human tissues, there has been a dearth of *in vivo* reports on circadian expression of clock genes [but see 7, 31, 32]. It has also remained unclear whether phase differences in clock gene mRNA oscillations between chronotype groups are large enough or robust enough to measure under non-lab conditions. Direct, real-time measurement of circadian rhythms is a necessary tool for examining how gene expression and/or genotype influences chronotype. Understanding the interaction between an individual’s genetic background, molecular clockwork, and environment is a critical gap in our knowledge of how the circadian clock affects human behavior.

The biggest obstacle to studying the molecular basis of individual circadian rhythms has been the lack of methods for non-invasive sampling of genetic data in sufficient quality and quantity to measure small changes in gene expression. Traditionally, measures of human circadian genes required blood tests or biopsies [33]. Alternatively, buccal swabs can be used but are generally poor candidates for RNA extraction due to ribonucleases present in saliva that rapidly degrade epithelial cell RNA [34]. This obstacle can now be overcome by making use of peripheral clock pacemakers. In recent studies, a new method for extracting RNA from human hair follicles allows one to measure the expression of human clock genes at multiple time intervals with a relatively non-invasive pulling of scalp hair [7, 32, 35]. In addition, using a simple mathematical model to estimate the cosine curve of oscillations in clock gene expression allows for the estimation of circadian oscillations from only 3 time-points of data [7]. This procedure facilitates large-scale studies of circadian gene expression and behavior.

We studied the expression of two principal clock genes, *Per3* and *Nr1d2*, following the methods of Akashi et al. [7]. Although *Per3* and *Nr1d2* are not components of the classical core negative feedback loop of the circadian clock, both genes give strong, cosine-fit, oscillatory signals and act as measurable markers in functioning circadian clocks [7]. *Per3* codes for the PER3 protein, which acts in a feedback loop as a repressor for *Clock* and *Bmal1* transcription. *Per3* is structurally similar to *Per1* and *Per2* and contains PAS domains that bind to cryptochrome 1 and 3 and many possible sights for phosphorylation [36, 37, 38]. *Nr1d2* codes for a nuclear hormone receptor protein that acts as a transcriptional repressor and may play a role in the regulation of carbohydrate and lipid metabolism [7, 39].

After identifying individuals of extreme morning and evening typologies, we analyzed expression patterns of *Per3* and *Nr1d2* to test for phase differences in clock gene expression between chronotype groups. From only three time points, we validate previous correlations between clock phase and chronotype using direct measures of circadian gene oscillations in hair follicle cells, a peripheral clock in humans, and estimate the phase difference between extreme morning and evening chronotypes.

## Methods

### Subjects

Thirty-six individuals from the faculty and undergraduate student population of Colgate University were selected for chronotype screening based on self- or peer-reported tendencies towards morningness or eveningness. All subjects completed the automated computer survey and genetic sampling (16 males, 20 females, age range 20–61). Fourteen of these individuals scored as extreme phenotypes; 7 were identified as ‘morning’ chronotypes and 7 as ‘evening’ chronotypes. Twenty-two scored as ‘moderate morning’, ‘moderate evening’ or ‘neither’. Informed consent was obtained from all individuals before samples were taken. All methods were developed in agreement with the Declaration of Helsinki; procedures and consent forms were approved by the Institutional Review Board at Colgate University (#FR-F13-07).

### Survey

An automated survey, including the Horne-Ostberg Morningness-Eveningness Questionnaire (MEQ) [15], was administered to each participant. The MEQ consists of nineteen questions that assess diurnal preference (i.e. timing of daytime activities, sleeping habits, hours of peak performance, times of maximum alertness, etc.). Individuals with high scores (>66) represent extreme morningness chronotypes while individuals with low scores (<46) represent extreme eveningness chronotypes. The correlation between MEQ scores and sleep-wake cycles has been validated in numerous previous studies [e.g. 40, 41].

### Circadian gene expression analysis

Hair follicle cells were sampled from the 14 participants with extreme chronotypes at three time points spaced 8 hours apart (8:00, 16:00, 24:00). Since the study was conducted on a single day, the downstream effect of disrupted sleep-wake cycles (possibly the 24:00 time point for morning-types and the 8:00 time point for evening-types) is anticipated to have minimal effects on oscillations in clock gene expression (unpublished data). RNA was purified from hair follicles by using an RNeasy Micro purification kit according to the protocol provided by Qiagen. The purified RNA was converted to cDNA via rt-PCR (TaqMan Gold rt-PCR, ABI). cDNA was quantified (Nanodrop) and the expression of clock genes was measured via quantitative PCR on an ABI 7900HT instrument (Applied Biosystems). qPCR analyses were performed in triplicate for two principal clock genes (*Per3, Nr1d2*) and the control gene, *18S*. The quantification of relative mRNA levels and standard errors was calculated using the ∆∆Ct method (ABI User Bulletin #2). Sample size for evening-types was reduced to n = 6 due to a missing time point for one individual at the *Nr1d2* locus in qPCR analyses of phase differences using both genes.

Differences in average gene expression between gender and age categories were tested using two-sample t-tests. Χ^2^ tests were used to compare age and gender differences in chronotype groups. Differences in gene expression levels across time and chronotype groups and time by chronotype interactions were tested using repeated measures ANOVAs. Correlations between MEQ scores, phase differences and wake time were analyzed with linear regressions. All statistical analyses were performed using SPSS software.

### Estimates of morningness-eveningness phase differences

To calculate phase differences between diurnal preference groups, three time points of mRNA level per gene per individual were used to estimate a cosine curve using a non-linear, least squares method with the following equation adapted from Akashi et al. [7]:

\[E(t) = A\cos \left\{ {\frac{{2\pi (t + \varpi )}}{{24}}} \right\} + c\]

with E(t) indicating gene expression level at time t, A indicating the amplitude of the cosine curve, ϕ indicating the phase, and C representing the offset value. To provide a control condition/training data set for this algorithm, we used gene expression data (nine time points) from four individuals from the previously published study to represent natural variation in circadian rhythms [7]. The phi values estimated from the model represent the phase difference between a control expression curve (representing the intermediate, ‘neither’ chronotype) and an individual’s curve (the ‘extreme’ phenotype). We used bootstrapping analyses to validate the control data set and the robustness of the estimation of phi values from extreme phenotypes. Phi values were averaged across individuals of similar extreme chronotypes and differences between the average morning versus evening chronotype values were tested using two-sample t-tests. We analyzed both genes separately and ran an additional test combining the expression data from both genes.

## Results

There were no significant differences in gender (χ^2^ = 2.80, p = 0.09) or age (college-age versus 35+ age, χ^2^ = 2.57, p = 0.11) represented in the two extreme (morning or evening) chronotype groups. Overall gene expression levels of *Per3* or *Nr1d2* did not differ by gender (*per3*: t = 0.08, p = 0.94; *Nr1d2*: t = 1.07, p = 0.29) or chronotype (*Per3*: t = 0.25, p = 0.80; *Nr1d2*: t = 0.61, p = 0.54).

**Figure 1:**
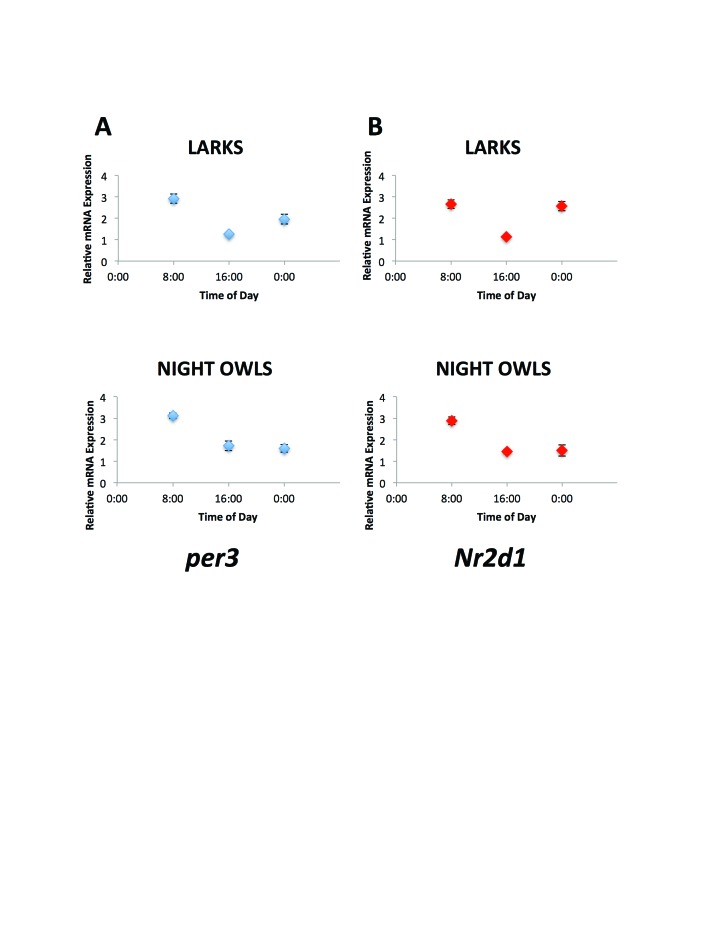
Relative mRNA levels (±SE) at three sampled time points for Per3 (A) and Nr1d2 (B). Patterns of relative gene expression varied between extreme morning and evening chronotypes but were similar across the two genes. On average, there was a 2.61-hour phase difference in Per3 expression (t = 2.58, p = 0.02) and a 3.08-hour phase difference in Nr1d2 expression (t = 2.91, p = 0.01) between the two chronotypes. Combining the data predicted from both genes, there was a 2.86-hour phase difference between extreme morning and evening chronotypes (t = 2.41, p = 0.03).

Relative gene expression levels differed across time points, and the interaction between chronotype group and time was significant for both genes (*Per3*: F_time_ = 24.73, p<0.001; F_timeXchronotype_ = 3.29, p = 0.05; *Nr1d2:* F_time_ = 21.8, p<0.001; F_timeXchronotype_ = 5.89, p=0.008 ). Patterns of relative gene expression varied between extreme morning and evening chronotypes (Figure 1) but were similar for the two genes, *Per3* or *Nr1d2*. On average, there was a 2.61-hour phase difference in *Per3* expression (t = 2.58, p = 0.02) and a 3.08-hour phase difference in *Nr1d2* expression (t = 2.91, p = 0.01) between the two chronotypes. Combining the data predicted from both genes, there was a 2.86-hour phase difference between extreme morning and evening chronotypes (t = 2.41, p = 0.03).

**Figure 2:**
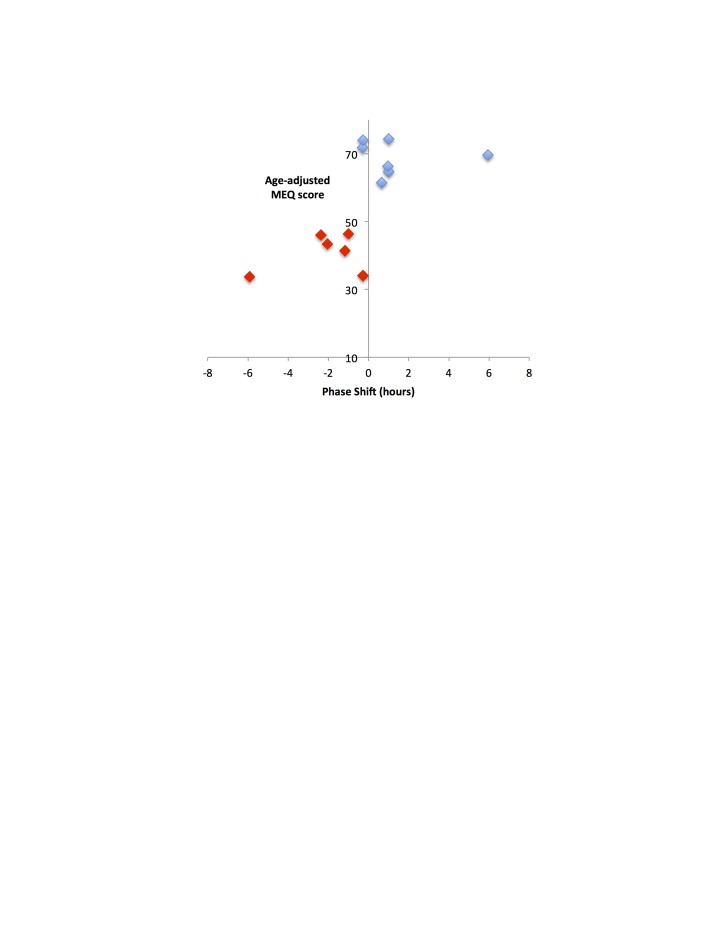
Clock phase predicts chronotype. Age-adjusted MEQ scores (y-axis) were significantly correlated with phase differences of individuals (r = 0.75, F = 13.85, p = 0.003). Red, extreme evening chronotypes; blue, extreme morning chronotypes.

**Figure 3:**
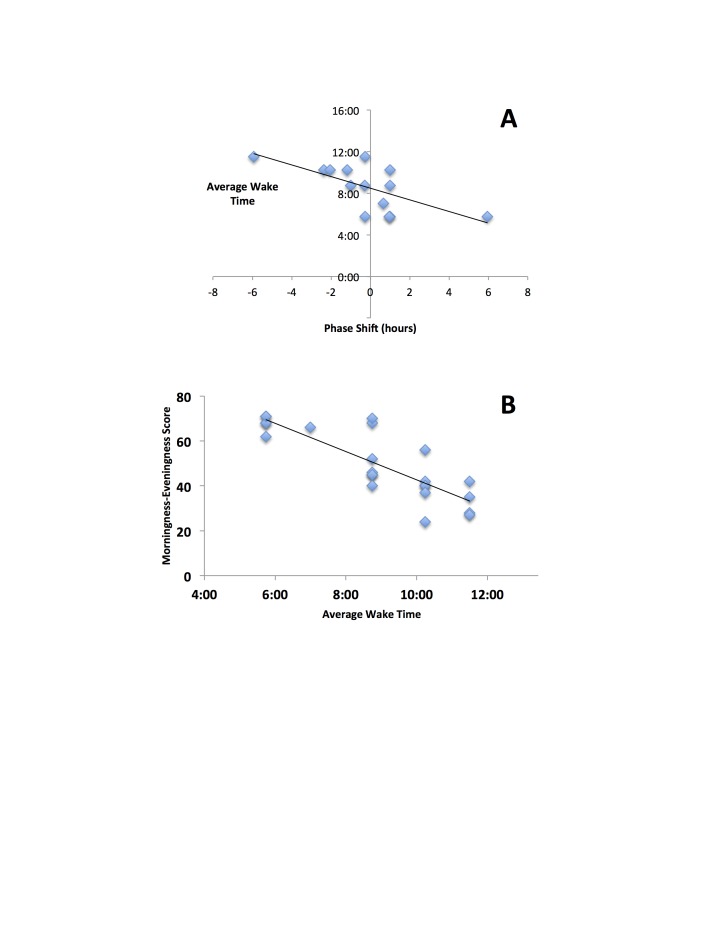
Relationships between wake-time and individual-specific clock phase and MEQ score. A: Average wake time in extreme chronotypes show a weak association with individual-specific phase differences (r = 0.66, F = 9.08, p = 0.011). B: Average wake times of all subjects (including non-extremes, n = 23) were correlated, as expected, with morningness-eveningness (MEQ) scores (r = 0.83, F = 46.33, p<0.001).

Age-adjusted MEQ scores were significantly correlated with phase differences of individuals (Figure 2; r = 0.75, F = 13.85, p = 0.003). Average wake time in extreme chronotypes showed a weaker association with individual-specific phase differences (Figure 3A; r = 0.66, F = 9.08, p = 0.011), but average wake times of all subjects (including non-extremes, n = 23) were correlated, as expected, with MEQ scores (Figure 3B; r = 0.83, F = 46.33, p < 0.001).

Bootstrapping analyses validated the use of control subjects to train our model. After selectively removing individual control subjects from each analysis, there were no significant differences in estimates of morning-evening phi values of test (extreme) subjects (for *Per3*, p = 0.36; for *Nd1r2*, p = 0.12; for both genes combined, p = 0.31).

## Discussion

The pioneering work of Brown and colleagues [1] on dermal-cell peripheral clocks was the first study to document phase differences in circadian gene expression in morning and evening types using fibroblast cultures maintained in laboratory conditions. However, the methods used in the study could not be easily translated into human behavior studies in a non-laboratory environment with large numbers of participants, as multiple skin biopsies are unpleasant and cell culture techniques expensive and laborious. The discovery of a non-invasive, efficient method of procuring peripheral clock gene expression from hair follicle cells [7, 32, 35] allows for direct measurement how circadian gene expression correlates with chronotype *in vivo*.

In this study, RNA extracted from hair follicle cells produce high quality total RNA of sufficient quantity for circadian gene expression analysis via quantitative rt-PCR. Our convenient, non-invasive collection methods closely follow those suggested by Akashi and colleagues [7] with similar success in RNA yield and quality from 10–20 scalp hairs. We demonstrate that extreme morning and evening chronotypes, designated by participant self-evaluation on a standard Morning-Eveningness questionnaire [15] have a significant phase shift in oscillation of circadian genes in the peripheral clock operating in hair follicle cells. Using only three data points per individual, we show that mRNA levels of both *Per3* and *Nr1d2* vary significantly over time and show consistent differences in phase between morning and evening types relative to 18S rRNA levels. Our results reveal that chronotype can be predicted from phase differences in oscillating mRNA. Individuals of particular chronotype groups had similar phase shifts; advanced phases occurred in morning-types, and delayed phases occurred in evening-types.

Our results are consistent with other studies demonstrating differences between morning and evening chronotypes in daily oscillations of body temperature, melatonin levels, circadian clock gene mRNA and proteins. Baehr and colleagues [42] report an approximate 2.1-hour phase difference in minimum body temperature between morning and evening types. In young and old subjects, the onset of melatonin production and core temperature rhythms occurs earlier in morning than in evening types [26, 29]. The phase angle for both of these variables (the interval between the phase marker and wake-time) appears to be longer in morning types. Although phase angle and peak levels of mRNA were not directly measured in this study, levels of clock gene mRNA were high in early morning hours, consistent with findings that peak period expression occurs prior to or near wake-time in other studies of clock gene expression in humans [7, 32, 35], mice [7] and horses [31]. As expected, average wake-time of individual participants was correlated with their morningness-eveningness score. We found a weaker correlation between average wake-time and clock phase, suggesting that average wake-time is not the only factor influencing the biological rhythms of extreme morning- and evening-types. One limitation of this study is that we calculate phase differences using only three time points estimate the circadian cosine curve. This approach has been validated by Akashi et al., 2010[7]; accuracy of cosine prediction in that study was highest with the sampling time interval of 8 h-8 h, which we used here.

In our study, individuals sharing extreme chronotypes varied in the magnitude of the phase difference in oscillating mRNA levels. Because we did not entrain subjects to a particular LD cycle for this experiment, individuals did not have synchronized sleep-wake cycles and our results represent natural (environmental and endogenous) variation in life-styles of the extreme chronotype groups. It is important to note that hair follicle cells represent a peripheral clock that is partially controlled by output from the suprachiasmatic nucleus (SCN) central pacemaker and there is expected to be a lag in output signals from the SCN. Although this lag is likely to be similar in morning-type and evening-type individuals, it is possible that peripheral clocks receive and process additional physiological inputs that may dictate differences between individuals, independent of light/dark cycles. The variation in gene expression profiles among subjects in this study thus represents the diverse environmental factors that affect life-styles and sleep-wake cycles of individuals, e.g. work/class demands, feeding schedules, etc.

Given the ‘field’ experimental design, the robustness of the phase differences between extreme chronotype groups underscores the biological basis of MEQ phenotypes. Measuring the oscillation of mRNA in peripheral clock genes from hair follicle cells provides a method for comparing the influence of specific environmental and/or endogenous genetic factors on clock-related behavior in future studies. Determining how clock gene polymorphisms and differences in rhythmic clock gene expression influence human activity and behavioral choices is key to understanding the effects of social jetlag, shift-work and other clock disruptions on human behavior.

## Conclusion

Analysis of *Per3* and *Nr1d2* mRNA levels from hair follicle cells under real-life conditions provides two valuable markers for estimating peripheral clock oscillations in human behavioral studies. The ease of sampling hair tissue and the robust phase differences between extreme chronotypes support the utility of this method in disentangling genetic versus environmental influences on human behavior. Given the importance of diurnal preference in the psychological and behavioral literature, a better understanding of the molecular basis of chronotype-specific behavior is necessary.

## Competing interests

The authors declare they have no competing interests.

## Author’s contributions

KKI conceived of the study, analyzed the data and drafted the manuscript. AF & DG collected the data and performed the gene expression assays. AA performed the mathematical estimates of phase data. AF, KJ, NB & KW assisted in drafting the manuscript. All authors approved of the final manuscript.
